# A commercial grain-free diet does not decrease plasma amino acids and taurine status but increases bile acid excretion when fed to Labrador Retrievers

**DOI:** 10.1093/tas/txaa141

**Published:** 2020-07-24

**Authors:** Renan A Donadelli, Julia G Pezzali, Patricia M Oba, Kelly S Swanson, Craig Coon, Jessica Varney, Christine Pendlebury, Anna K Shoveller

**Affiliations:** 1 Animal Biosciences Department, University of Guelph, Guelph, ON, Canada; 2 Department of Animal Sciences, University of Illinois at Urbana-Champaign, Urbana, IL; 3 Four Rivers Kennel, LLC., Walker, MO; 4 Research and Innovation Department, Champion Petfoods LP, Edmonton, AB, Canada

**Keywords:** grain-free dog food, large-breed dogs, pulses, sulfur amino acids

## Abstract

Grain-free diets tend to have greater inclusions of pulses in contrast to grain-based diets. In 2018, the Food and Drug Administration (FDA) released a statement that grain-free diets may be related to the development of canine dilated cardiomyopathy (DCM). However, all dog foods met regulatory minimums for nutrient inclusion recommended by the Association of American Feed Controls Official. In some FDA case reports, but not all, dogs diagnosed with DCM also had low concentrations of plasma or whole blood taurine; thus, we hypothesized that feeding these diets will result in reduced taurine status from baseline measures. The objective of this study was to determine the effects of feeding a grain-free diet to large-breed dogs on taurine status and overall health. Eight Labrador Retrievers (four males and four females; Four Rivers Kennel, MO) were individually housed and fed a commercial complete and balanced grain-free diet (Acana Pork and Squash formula; APS) for 26 wk. Fasted blood samples were collected prior to the start of the trial (baseline; week 0) and at weeks 13 and 26 for analyses of blood chemistry, hematology, plasma amino acids, and whole blood taurine. Urine was collected by free catch at weeks 0 and 26 for taurine and creatinine analyses. Fresh fecal samples were collected at weeks 0 and 26 for bile acid analyses. Data were analyzed using the GLIMMIX procedure with repeated measures in SAS (v. 9.4). Plasma His, Met, Trp, and taurine and whole blood taurine concentrations increased over the course of the study (*P* < 0.05). Urinary taurine to creatinine ratio was not affected by diet (*P* > 0.05). Fecal bile acid excretion increased after 26 wk of feeding APS to dogs. Despite the higher fecal excretion of bile acids, plasma and whole blood taurine increased over the 26-wk feeding study. These data suggest that feeding APS, a grain-free diet, over a 26-wk period improved taurine status in Labrador Retrievers and is not the basis for the incidence of DCM for dogs fed APS. Other factors that may contribute to the etiology of DCM should be explored.

## INTRODUCTION

In 2018, the Food and Drug Administration (FDA) released a report warning about a possible link between feeding dogs (specifically large male dogs) grain-free diets and the development of dilated cardiomyopathy (DCM); efforts to understand the potential link continues. Grain-free diets are loosely defined as dog foods that contain no grains and instead contain peas, lentils, other leguminous seeds (pulses), and/or potatoes in various forms (whole, flour, protein, etc.) as the main ingredient (listed within the first 10 ingredients in the ingredient list, before vitamins and minerals). Legumes, specifically pulses, have greater protein [National Research Council (NRC), 2006; Singh, 2017] and fiber (especially soluble fibers and oligosaccharides) content (Carciofi et al., 2008) in contrast to grains (Alvarenga and Aldrich, 2019; Pezzali et al., 2020). While these characteristics may help to increase dietary protein content and aid in gut health and weight management (Choct, 2009; German et al., 2010; Flanagan et al., 2017; Kroger et al., 2017), pulses contain lower concentrations of sulfur amino acids (SAA) and may cause imbalances in a diet’s amino acid (AA) profile. Other limitations in pulses’ nutrient composition include the complete lack of taurine and L-carnitine content (NRC, 2006; Hall et al., 2017; Mansilla et al., 2019), which are required to support osmoregulatory balance and fatty acid oxidation, respectively.

There is a known relationship between low taurine status and DCM. One common parameter reported to the FDA was low plasma and whole blood taurine concentrations in dogs with DCM. In Newfoundland dogs with DCM, taurine supplementation was reported to reverse the condition (Fascetti et al., 2003). Additionally, when taurine was supplemented to Golden Retrievers with DCM, there was an improvement in health and, in some cases, the disease was resolved (Belanger et al., 2005). Similarly, other cases of taurine supplementation in DCM-diagnosed dogs lead to similar outcomes (Kittleson et al., 1997; Backus et al., 2006; Kaplan et al., 2018; Adin et al., 2019).

Taurine is a β-amino sulfone that is not used for protein synthesis but remains in animal tissues as a free AA (Brosnan and Brosnan, 2006). Dogs are able to synthetize taurine if sufficient dietary SAA (Met and cyst(e)ine) are provided (Torres et al., 2003). As a result, despite being involved in several metabolic roles (Huxtable, 1992; Sanderson, 2006), taurine is not considered an essential nutrient for dogs [NRC, 2006; Association of American Feed Controls Official (AAFCO), 2018]. One of the primary roles of taurine in dogs is the conjugation of bile acids (BA; Czuba and Vessey, 1981). After primary BA aid in fat digestion, they are reabsorbed and transported back to the liver (Dawson and Karpen, 2015); however, some dietary components may affect BA recirculation. For example, BA recirculation can be affected by fibers (Kritchevsky, 1978) and high fat content (Bravo et al., 1998; Herstad et al., 2017; Herstad et al., 2018). Since taurine is conjugated to BA, loss could occur through feces due to reduced enterohepatic recirculation of BA (Hickman et al., 1992; Ajouz et al., 2014; Dawson and Karpen, 2015) and result in a reduction in taurine status. Moreover, taurine is also lost through urinary excretion, with dietary supplementation causing an increase in excretion (Park et al., 1989). As such, taurine balance can be assessed by measuring plasma and whole blood taurine, fecal BA excretion, and urinary taurine excretion.

To date, there are few published studies evaluating the effects of a commercial grain-free diet and taurine status in healthy large-breed dogs, and most pet food companies do not publish AAFCO feeding studies. Thus, the objective of this study was to evaluate the effects of feeding a commercial grain-free dog food to Labrador Retrievers on apparent total tract digestibility (ATTD), stool quality, blood chemistry, hematology, plasma AA and taurine concentrations, and BA excretion during a 26-wk feeding trial that followed AAFCO Canine Feeding protocols. We hypothesized that dogs fed a commercial complete and balanced grain-free diet with adequate concentrations of SAA and taurine would: 1) have normal blood chemistry and hematology; 2) normal plasma AA concentrations; 3) reduced blood and plasma taurine concentrations; and 4) a higher fecal excretion of primary and secondary BA and urinary taurine.

## MATERIALS AND METHODS

The Institutional Animal Care and Use Committee (IACUC) of Four Rivers Kennel (Walker, MO) approved this study (IACUC number FRK-14). The study was conducted at Four Rivers Kennel from October 18, 2018 to April 18, 2019. The study used monadic feeding with repeated measures and followed the recommendations of the AAFCO (2018) feeding protocol to support a complete and balanced adult maintenance claim. Additional outcomes focused on taurine metabolism were added to test the hypotheses.

### Dog Food and Feeding Trial

Eight Labrador Retriever dogs (four intact males and four intact females, average age = 6.0 ± 2.1 yr) were housed individually (1.83 × 1.22 m) and fed a grain-based commercial control diet (CTL; MFA Gold N Pro, MFA Inc., Columbia, MO; Table 1) for the 26-wk washout prior to the start of the study. Kennel temperature was held between 13 and 29 ºC and humidity was dependent on the weather. Depending on weather conditions, dogs had socialization with other dogs for up to 6 h daily in outside runs (12.2 × 12.2 m).

**Table 1.** Analyzed nutrient composition of kennel and test diets

Composition, %	APS^*a*^	CTL^*b*^
Moisture	8.40	6.50
	% dry matter	
Crude protein	37.81	30.89
Crude fat	18.78	16.79
Ash	8.06	9.97
Nitrogen free extract^*c*^	23.95	28.66
Total dietary fiber	11.40	13.60
Insoluble fiber	9.50	11.90
Soluble fiber	1.90	1.70
Arginine	2.25	1.72
Histidine	0.79	0.55
Isoleucine	1.19	1.03
Leucine	2.31	2.32
Lysine	2.31	1.15
Methionine	0.55	0.43
Methionine + cystine	0.85	0.99
Phenylalanine	1.32	1.20
Threonine	1.23	1.05
Valine	1.51	1.43
Tryptophan	0.30	0.22
Methionine:cystine	1.83	0.77
Taurine	0.14	0.07
Cholesterol, mg/100g	140	98.5

^*a*^APS: test diet, Acana port, and squash. Ingredient composition: deboned pork, pork meal, whole lentils, pork liver, pork fat, whole peas, lentil fiber, pea starch, butternut squash, pollock oil, natural pork flavor, pork cartilage, pumpkin, salt, mixed tocopherols, zinc proteinate, dried kelp, calcium pantothenate, taurine, freeze dried pork liver, copper proteinate, chicory root, turmeric, dried *Lactobacillus acidophilus* fermentation product, dried *Bifidobacterium animalis* fermentation product, and dried *Lactobacillus casei* fermentation product. Diet formulated to meet the requirement of all life stages (AAFCO, 2018).

^*b*^CTL: kennel diet, MFA Gold N Pro. Ingredient composition: poultry byproduct meal, ground corn, corn distillers dried grain with solubles, pearled barley, poultry fat, porcine meal, dried plain beet pulp, poultry liver, flavors, flax seeds, and minerals and vitamins. Diet formulated to meet the requirement of adult dogs at maintenance (AAFCO, 2018).

^*c*^Nitrogen-free extract calculated as subtracting crude protein, crude fat, total dietary fiber, and ash from the dry matter.

After the 26-wk washout period (baseline; week 0), dogs were fed the treatment grain-free diet [Acana Pork and Squash formula (APS), Champion Petfoods, Auburn, KY]. Males were fed 700 g/d and females were fed 550 g/d; feeding amounts were based on their energy requirements from the previous 26 wk. Clean, fresh water was available ad libitum. Any remaining food was weighed daily to calculate total feed intake.

### Blood Collection and Analyses

At weeks 0, 13, and 26, blood samples were collected between 9:00 and 10:00 a.m. after dogs were fasted for 24 h. Blood was sampled using two lithium heparin tubes (4 mL per tube; BD 367884) and one ethylenediaminetetraacetic acid tube (2 mL; BD 367841). After blood collection, tubes were gently inverted 8–10 times and stored in a refrigerator (approximately 2 ºC) before analysis. One of the lithium heparin tubes from each dog was shipped with cool packs to the University of California, Davis Amino Acid Laboratory (Davis, CA) for whole blood taurine analysis. The other two lithium heparin tubes were centrifuged at 1,500 × *g* for 10 min at 4 ºC and the plasma (approximately 1.2 mL) was transferred to a cryovial. One of the cryovials was shipped to the University of California, Davis Amino Acid Laboratory (Davis, CA) for the determination of plasma AA according to Kim et al. (1995). All samples that were shipped to a laboratory were sent with cool packs and were received by the laboratories on the next day to decrease sample degradation. Another 0.25 mL of plasma was collected and refrigerated for blood chemistry analysis (Abaxis VetScan2) at the Four Rivers Kennel Laboratory (Walker, MO). The ethylenediaminetetraacetic acid tube was stored refrigerated (2 ºC) until blood hematology was determined at Four Rivers Kennel Laboratory using an Abaxis HM5 (Abaxis, Union City, CA).

### Fecal and Urine Collection and Analyses

During the 5-d fecal collection phase, feces were scored in the morning of each collection day on a five-point scale (1 being liquid diarrhea and 5 being feces that are fully formed, very firm, with little moisture, hard, and crumbles easily) and collected in a specimen cup and frozen at −20 ºC. After the 5 d of collection, the content of the specimen cups for each dog was homogenized using a hand blender and kept frozen. A subsample was sent to Eurofins Scientific Inc. (Des Moines, IA) to be analyzed for dry matter and macronutrient concentration. Urine samples were collected once for each dog during the 5 d of fecal collection before feeding by free catch in a clean ladle. After being collected, the urine samples were refrigerated (2 ºC) for approximately 1 h before they were centrifuged at 1,000 × g for 20 min at 4 ºC. After centrifugation, a 1-mL aliquot was separated and analyzed for taurine (University of California Davis Amino Acid Laboratory) and a 3-mL aliquot for creatinine (University of California Davis Central Laboratory) concentrations. In addition, a fresh fecal sample (approximately 20 g) were collected and frozen at −80 ºC at baseline and week 26 for subsequent fecal BA analyses (Gastrointestinal Laboratory, Texas A&M University, College Station, TX).

### Apparent Total Tract Macronutrient Digestibility Estimation

During the last 6 d of the feeding trial, all dogs received a capsule containing 2 g of titanium dioxide with their morning feeding for the estimation of apparent total tract macronutrient digestibility. Fecal samples were collected during the last 5 d of week 26, homogenized, and frozen prior to other sample preparation. Fecal samples were analyzed for moisture (AOAC 930.15), crude protein (AOAC 990.03), acid-hydrolyzed fat (AOAC 954.02), ash (AOAC 942.05), neutral detergent fiber (Ankom NDF), acid detergent fiber (Ankom ADF 05-03), and titanium dioxide (Myers et al., 2004; Alvarenga et al., 2019). ATTD was estimated using the equation below:

$$\begin{array}{l} ATTD=\left(1-\frac{TD\;\times \;NF}{TF\;\times \;ND}\right)\;\times \;100 \end{array}$$

where TD is the titanium dioxide content in the diet as a percentage, NF is the nutrient concentration in the feces as a percentage, TF is the titanium dioxide content of the feces as a percentage, and ND is the nutrient concentration in the diet as a percentage.

### Statistical Analysis

This study was designed as a monadic feeding study and data were analyzed as repeated measures with time considered as a fixed effect. The GLIMMIX procedure (SAS v. 9.4, The SAS Institute, Cary, NC) and a repeated measures model were used to analyze the data. Dog was considered the experimental unit and dog nested within week was considered the random effect. Means were separated by Fisher’s LSD considering an alpha of 0.05. Data reported in tables are expressed as least square means (±SEM).

## RESULTS AND DISCUSSION

The current study sought to describe the physiological effects of feeding a commercial grain-free formula on taurine status and overall health of Labrador Retriever dogs. Due to the FDA reports on the possible association between increased presentation of DCM cases and diet fed to those dogs, this study collected key data surrounding SAA metabolism and specifically taurine. The dogs started the trial with plasma Met concentrations above the upper limit of the reference range (93.5 nmol/mL, range 57 ± 2 nmol/mL; Table 2), yet plasma Met concentrations increased over time (*P* < 0.05) and plasma cystine was not affected (*P* > 0.05). This suggests that diet and protein turnover may have maintained plasma Met concentrations. Met is used as a methyl donor in the production of Cys and other sulfur-containing compounds. Accurate quantification of total Cys requires biological samples to be reduced and prepared differently than the methodology used to measure the other AA (Shoveller et al., 2003; Shoveller et al., 2004). Because the analyses used does not quantify total cyst(e)ine and cystine is not considered an accurate measure of total cyst(e)ine, we have chosen not to discuss these results. Additionally, Cys is known to be reactive and losses occur rapidly after blood is collected (Torres et al., 2004). In the present study, samples were shipped overnight to UC Davis Amino Acid Laboratory in cool packs; however, this may not have been enough to prevent sample degradation and it is a limitation of this work. Thus, Cys and cystine values here reported must be considered with caution. Future studies should consider different methods to measure total cyst(e)ine (Torres et al., 2004) and also quantify homocysteine and glutathione to better understand the regulation of SAA metabolism in dogs.

**Table 2.** Whole blood taurine and plasma amino acid concentrations of Labrador Retrievers fed a grain-free diet for 26 wk (*n* = 8)

Amino acid, nmol/mL	Week			SEM	*P*-value
	0	13	26		
L-alanine	314^b^	267^c^	399^a^	15.8	<0.0001
L-arginine	66.8	82.3	63.0	29.5	0.8865
L-a-amino-n butyric acid	70^b^	125^ab^	193^a^	27.3	0.0159
L-asparagine	34.0	29.9	35.8	6.03	0.7838
L-aspartic acid	24.5^b^	33.9^b^	69.4^a^	9.26	0.0062
L-citrulline	30.4	26.1	29.3	11.1	0.9594
Cystathionine	33.5	60.8	66.4	13.1	0.1902
L-cystine	4.13	2.60	3.56	0.82	0.432
L-glutamic acid	46.5	24.0	23.2	15.2	0.482
L-glutamine	142	240	181	74.3	0.6485
Glycine	215^b^	595^a^	494^a^	55.3	0.0003
L-histidine	161^b^	185^b^	306^a^	35.9	0.0216
1-methyl-l-histidine	43.0	85.4	76.2	15.2	0.1412
3-methyl-l-histidine	8.3^b^	12.2^ab^	13.8^a^	1.41	0.0321
L-isoleucine	30.4	31.0	42.9	11.1	0.6758
L-leucine	75.4^b^	82.0^b^	144.0^a^	13.3	0.0025
L-lysine	114^b^	132^b^	234^a^	13.9	<0.0001
L-methionine	94^b^	128^b^	230^a^	24.0	0.0017
L-ornithine	38.8^b^	54.8^b^	86.6^a^	7.36	0.0006
L-phenylalanine	39.0^b^	39.7^b^	88.2^a^	9.91	0.0025
L-proline	90.8	90.8	138.5	18.1	0.123
Hydroxy-l-proline	72.8^b^	86.1^b^	251.4^a^	27.7	0.0002
L-serine	82.1	64.7	121.6	22.9	0.2243
Taurine	107^c^	157^b^	192^a^	11.4	0.0001
L-threonine	142^ab^	128^b^	199^a^	20.7	0.0557
Tryptophan	105^b^	169^ab^	226^a^	29.5	0.0291
L-tyrosine	39.4^c^	58.8^b^	73.4^a^	3.33	<0.0001
L-valine	99.0	81.2	122.1	27.9	0.5898
Taurine (whole blood)	186^b^	204^b^	295^a^	20.2	0.0021

^abc^Means in a row with different superscripts differ, *P* < 0.05.

Whole blood concentrations of taurine tend to be more accurate and is an indication of intracellular taurine content (Pacioretty et al., 2001) in contrast to plasma concentrations. One possible explanation for these differences is that taurine can be released from white blood cells and platelets during clotting (Torres et al., 2004; Kaplan et al., 2018); therefore, plasma taurine content is more variable than in whole blood. In the current study, whole blood taurine concentrations were above the upper confidence limit of 250 nmol/mL (Kaplan et al., 2018) only at week 26. This is an indication that whole-body taurine status increased in Labrador Retriever dogs consuming APS (Table 2). The increase in taurine over time may be due to the higher concentrations of taurine in the APS diet compared to the CTL (0.14 vs. 0.07, respectively). Similarly, Pezzali et al. (2020) reported an increase in plasma taurine concentration in Beagle dogs fed a grain-free diet supplemented with taurine over a 28-d feeding trial. Although whole blood and plasma concentrations of taurine are commonly used to evaluate taurine deficiency in dogs in a clinical setting, it is as important to consider the urinary and fecal losses. Whole-body taurine pool size can be regulated through urinary reabsorption/excretion (Chesney et al., 2010). Urinary concentrations of taurine, presented as taurine:creatinine, were similar (*P* > 0.05) between weeks 0 and 26 and suggests that dogs were not taurine deficient (Table 3). In contrast, Pezzali et al. (2020) reported that Beagle dogs fed a grain-free diet and supplemented with taurine had a higher urinary taurine:creatinine excretion at days 14 and 28 compared to baseline measurements. This difference could be related to the higher taurine concentration of the diets fed in the study by Pezzali et al. (2020) compared to our diets (0.34% vs. 0.14%, respectively) but suggests that both grain-free diets met or exceeded the metabolic requirements for taurine.

**Table 3.** Urine taurine, creatinine, and taurine:creatinine ratio and fecal bile acids and cholesterol of Labrador Retrievers fed a grain-free diet for 26 wk (*n* = 8)

Parameter	Week		SEM	*P*-value
	0	26		
	Urine			
Taurine:creatinine	0.25	0.28	0.07	0.7760
	Fecal bile acids, µg/mg			
Cholic acid	0.055^b^	0.142^a^	0.024	0.0193
Chenodeoxycholic acid	0.008^b^	0.037^a^	0.008	0.0300
Lithocholic acid	0.74	0.94	0.07	0.0622
Deoxycholic acid	3.45^b^	5.52^a^	0.58	0.0237
Ursodeoxycholic acid	0.0065	0.0066	0.0004	0.8358
Primary bile acids	0.063^b^	0.179^a^	0.027	0.0078
Secondary bile acids	4.19^b^	6.46^a^	0.64	0.0255
Total bile acids	4.26^b^	6.64^a^	0.65	0.0208
Cholesterol	0.98	1.1	0.079	0.2860
	Fecal bile acids, % of total			
Cholic acid	1.33	2.2	0.37	0.1154
Chenodeoxycholic acid	0.18	0.73	0.19	0.0671
Lithocholic acid	18.08^a^	14.67^b^	1.05	0.0367
Deoxycholic acid	80.23	82.3	1.22	0.2515
Ursodeoxycholic acid	0.17^a^	0.11^b^	0.017	0.0204
Primary bile acids	1.51	2.93	0.51	0.0682
Secondary bile acids	98.49	97.07	0.51	0.0682

^ab^Means in a row with different superscripts differ, *P* < 0.05.

In addition to urinary losses, fecal loss of taurine occurs when the BA are not reabsorbed through the enterohepatic circulation (Hickman et al., 1992; Ajouz et al., 2014; Dawson and Karpen, 2015). In the dog, most of the primary BA (cholic acid and chenodeoxycholic acid) are synthesized from cholesterol and conjugated with taurine (Czuba and Vessey, 1981; Imamura et al., 2000) rather than glycine (Zhang et al., 2016) before being secreted into the small intestine. Hamsters who consume greater dietary taurine experienced increased BA secretion (Bellentani et al., 1987); while this is yet to be measured in dogs, dietary taurine supplementation could also lead to an increase in BA secretion in dogs; however, it is generally accepted that the supplementation of taurine will lead to excretion in the urine (Chesney et al., 2010). If the BA are not reabsorbed, taurine can be deconjugated by the commensal bacterial enzyme BA hydrolase present in the terminal ileum and colon (Long et al., 2017). The unconjugated primary BA can be converted into secondary BA by *Clostridium* (cholic acid converted to deoxycholic acid), *Eubacterium* (chenodeoxycholic acid converted to lithocholic acid and ursodeoxycholic acid), and other bacterial taxa present in the colon, through the enzyme 7 α-dehydroxylase (Doerner et al., 1997; Benno et al., 2001; Ridlon et al., 2006). As such, fecal concentrations of BA may be used as a tool to evaluate fecal losses of taurine (Anantharaman-Barr et al., 1994).

In the current study, fecal concentrations of total BA increased from baseline and, at week 26, were 1.56 times greater (Table 3). This was due to a higher (*P* < 0.05) excretion of both primary (week 0 = 0.063 vs. week 26 = 0.179 µg/mg) and secondary (week 0 = 4.19 vs. week 26 = 6.64 µg/mg) BA. The increase in primary BA was a result of greater excretion (*P* < 0.05) of both cholic acid (week 0 = 0.055 vs. week 26 = 0.142 µg/mg) and chenodeoxycholic acid (week 0 = 0.008 vs. week 26 = 0.037 µg/mg). Pezzali et al. (2020) also observed a greater excretion of primary BA due to the excretion of cholic acid in Beagle dogs fed a grain-free diet; however, the excretion of chenodeoxycholic acid was not a factor. The excretion of BA can be affected by diet (Herstad et al., 2018); thereby, some dietary components (e.g., dietary fibers and fats) in the APS diet may play a role in the excretion of BA. The higher fecal primary BA concentrations after 26 wk of APS consumption may be due to the higher dietary fat content of the APS diet (week 0 = 16.8% vs. week 26 = 18.8%; Table 1), which would increase BA secretion (Bravo et al., 1998). An increase in BA excretion has been previously reported when dogs were fed a high minced beef diet compared to a commercial dry food as a response to different dietary fat levels (33.1% vs. 16.33%, respectively; Herstad et al., 2017, 2018).

In addition to the dietary fat content, dietary fibers may also bind BA in the small intestine and further increase BA excretion (Kritchevsky, 1978). Moreover, the fermentation of dietary fibers in the large intestine may lower the luminal pH and bacterial 7 alpha-dehydroxylase activity, reducing the conversion of primary BA to secondary BA (Bingham, 2000). Unfortunately, products of fermentation (e.g., short-chain fatty acids) and fecal pH were not analyzed in the present study and should be considered in future research. Despite the total dietary fiber of APS being less than CTL (11.4% vs. 13.6%; Table 1), the oligosaccharide content (not measured) likely was higher in APS due to the higher inclusion of pulses (Bednar et al., 2001; de Oliveira et al., 2012). Dietary oligosaccharide content contributes to an increase in bacterial fermentation in the gut (Felix et al., 2013). Ko and Fascetti (2016) also reported a higher excretion of BA when dogs were fed a purified diet with beet pulp (a moderately fermentable fiber). Although both this study and Ko and Fascetti (2016) reported low soluble fiber content for the experimental diets, there was an increase in BA excretion when dogs were fed APS for 26 wk. However, it is noteworthy that both plasma and whole blood taurine concentrations were improved over time for dogs fed APS and urinary taurine:creatinine was not affected by the diet. Therefore, even though greater fecal losses of BA occurred, this did not affect taurine status as supported by plasma and whole blood taurine concentrations and urinary taurine:creatinine throughout the duration of the study.

Secondary BA also increased from the baseline (week 0 = 4.19 vs. week 26 = 6.46 µg/mg), where deoxycholic acid was the major contributor (*P* < 0.05; week 0 = 3.45 vs. week 26 = 5.52 µg/mg). The rise in deoxycholic acid concentration, rather than that of lithocholic acid and ursodeoxycholic acid, indicates a greater ability of gastrointestinal bacteria to transform cholic acid rather than chenodeoxycholic acid. This could be due to substrate (cholic acid vs. chenodeoxycholic acid) availability, microbial populations present in the colon, or other unknown factors. Likewise, in a previous study, secondary BA and deoxycholic acid concentrations increased after dogs consumed a high minced beef diet for 7 wk compared to dogs fed a commercial dry food (Herstad et al., 2018). Unfortunately, microbiota population shifts due to the consumption of the grain-free diet were not analyzed in the present study. However, our results are different from Pezzali et al. (2020), who did not report a greater excretion of secondary BA due to deoxycholic acid in Beagle dogs fed a grain-free diet. Thus, a combination of factors in the APS diet, such as higher dietary fat content and soluble fiber concentrations, may have stimulated the higher concentrations of both fecal primary and secondary BA (Table 3).

We also observed a decrease in ursodeoxycholic acid and lithocholic acid as the percentage of total BA [ursodeoxycholic acid: week 0 = 0.2% vs. week 26 = 0.1% (*P* = 0.02); lithocholic acid: week 0 = 18.1% vs. week 26 = 14.7% (*P* = 0.04)]. In addition to BA concentration, primary and secondary BA as a proportion of total BA were not affected over time (*P* > 0.05; Table 3). The level of hydrophobicity of BA is positively associated with its cytotoxic potential (BA hydrophobicity scale: ursodeoxycholic acid < cholic acid < chenodeoxycholic acid < deoxycholic acid < lithocholic acid; Hofmann, 1999), with deoxycholic acid and lithocholic acid known to induce oxidative damage of DNA in vitro (Booth et al., 1997; Bernstein et al., 1999; Glinghammar, 2002; Payne, 2008; Rosignoli et al., 2008). In contrast, ursodeoxycholic acid is believed to have chemoprotective potential (Alberts et al., 2005; Akare et al., 2006). For this reason, a lower percentage of fecal lithocholic acid after 26 wk of consumption of APS may suggest a beneficial shift. However, the reduction in fecal ursodeoxycholic acid percentage may not be desirable. Our results are different from Pezzali et al. (2020) who observed a greater excretion of primary BA as a proportion of total BA and a lower excretion of secondary BA (deoxycholic acid + lithocholic acid) in Beagle dogs fed a grain-free diet. However, similar to the present study, Pezzali et al. (2020) also reported a lower excretion of lithocholic acid as a proportion of total in Beagle dogs fed a grain-free diet (information regarding ursodeoxycholic acid was not reported).

Fasting plasma concentrations (24 h fast) of Arg, Ile, Thr, and Val did not change over the course of the trial (*P* > 0.05; Table 2). In contrast, plasma concentrations of His, Leu, Lys, Met, Phe, and Trp increased from week 0 to 26 (*P* < 0.05; Table 2), suggesting that these AA were over requirement and drove increased protein turnover. Plasma AA concentrations are generally tightly controlled, increasing after consuming a meal and subsequently decreasing to fasting concentrations. Concentrations of Asp, 3-methyl-L-histidine, Leu, and Lys increased over time (*P* < 0.05), suggesting that there was greater protein turnover as the trial progressed and increasing the rate of protein turnover. This is supported by the increase in 3-methyl-L-histidine, which is a marker for protein breakdown (Chinkes, 2005; Holm and Kjaer, 2010) and is further supported by the increase over time of blood urea nitrogen, which is an indication of AA catabolism and subsequent ureagenesis. Blood urea nitrogen is associated with dietary protein and is likely due to the greater protein content of the test diet as compared to the baseline diet. Increased circulating indispensable AA and blood urea nitrogen are positively related to dietary protein intake (Kang et al., 1987). Future studies should consider dynamic measures of protein synthesis, such as rates of protein synthesis, or static measures over time, such as body composition by dual X-ray absorptometry or quantitative magnetic resonance imaging, to more accurately reflect the impact of AA status of dogs fed grain-free formulas.

Despite differences in plasma AA and fecal BA, during the study, the stool of dogs fed APS were considered ideal, with an average fecal score of 3.12 ± 0.13. Dry matter, crude protein, and crude fat ATTD were 86.6%, 90.5%, and 95.4%, respectively. The current study observed similar digestibility values as compared to Pezzali and Aldrich (2019; dry matter: 86.6% vs. 85.8%; crude protein: 90.5% vs. 87.2%; crude fat: 95.4% vs. 93.6%, respectively) who also fed a grain-free diet to dogs (38.0% crude protein, 12.5% crude fat, 3.85% insoluble fiber, 6.22% soluble fiber, 4.33% ash; ingredients: hydrolyzed pork protein, white potato, green peas, tapioca starch, minerals and vitamins, menhaden fish oil, taurine, antioxidant, chicken fat, flavor enhancer, and titanium dioxide) for a period of 28 d. Small differences between these two studies may be related to differences in breed (Labrador Retrievers vs. Beagles), environment, analytical variation, ingredient, and nutrient composition.

In the present study, dry matter and crude protein digestibilities were higher (dry matter: 86.6% vs. 79.6%, crude protein: 90.5% vs. 85.3%, respectively, for this study and Chiofalo et al., 2019) than what Chiofalo et al. (2019) reported for Labrador Retrievers fed a grain-free diet (39.24% crude protein, 18.69% crude fat, 11.59% total dietary fiber, 7.91% ash; ingredients: fresh grass-fed lamb, dehydrated lamb meat, potatoes, dried whole eggs, fresh herrings, dehydrated herring, chicken fat, herring oil, vegetable pea fiber, dried carrots, sun-cured alfalfa meal, inulin, fructooligosaccharinde, mannan-oligosaccharides, dehydrated blueberry, dehydrated apple, dehydrated pomegranate, dehydrated sweet orange, dehydrate spinach, psyllium seed husk, currant powder, salt, brewers dried yeast, turmeric, glucosamine, chondroitin sulfate, and tagete flower extract). Similar to Chiofalo et al. (2019), Meyer et al. (1999) reported lower values for crude protein and fat digestibilities (86.3% and 93.6%, respectively) in Labrador Retrievers fed a commercial diet (29.4% crude protein, 13.8 crude fat, 2.84% insoluble fiber, 0.55% soluble fiber, and 8.3% ash) compared to the present study. However, to truly understand AA digestibility, ileal digesta needs to be collected and corrected for diet-specific endogenous losses. As demonstrated by Johnson et al. (1998), nitrogen ATTD is numerically higher than ileal digestibility; similar results were also reported by Murray et al. (1998) and Hendrix and Sritharan (2002). A precision-fed cecectomized rooster assay reported (Oba and Swanson, 2019) that APS ileal digestibility was greater than 80% for most indispensable AA, with some greater than 90% (Oba and Swanson, 2019); however, the authors did not report which indispensable AA had digestibility lower than 80%.

Although some differences were observed over time, all the hematological parameters, with one exception, were within reference range for this cohort of dogs (Table 4). Mean corpuscular hemoglobin concentration was below reference range minimums on weeks 0 and 13 (29.9 vs. 30.5 10^3^/mm^3^, respectively; reference range: 31.0–39.0 g/dL) but within the reference range on week 26 (32.1 g/dL). While within reference range, alanine aminotransferase, alkaline phosphatase, and glucose decreased over time (Table 5; *P* < 0.05). Despite some analyzed parameters being affected over the course of the trial, it is unclear whether these changes have biological significance. In the context of AAFCO regulatory support, the response in serum biochemistry over 26 wk meet criteria for a complete and balanced adult maintenance claim.

**Table 4.** Hematology of Labrador Retrievers fed a grain-free diet for 26 wk (*n* = 8)

Blood parameter	Unit	Week			SEM	*P*-value
		0	13	26		
WBC	10^3^/mm^3^	8.79^b^	10.33^b^	13.71^a^	1.15	0.0188
RBC	10^6^/mm^3^	8.24	8.19	8.47	0.15	0.379
Hemoglobin	g/dL	16.3^b^	16.5^ab^	17.3^a^	0.31	0.0636
Hematocrit	%	54.6	54.2	54.1	1.16	0.9573
MCV	fL	66.4	66.3	63.8	1.05	0.1613
MCH	pg	19.8^b^	20.2^ab^	20.4^a^	0.20	0.0721
MCHC	g/dL	29.9^b^	30.5^b^	32.1^a^	0.34	0.0005
Platelets	10^3^/mm^3^	265	314	308	38.2	0.6163
Lymphocytes	10^3^/mm^3^	2.05^a^	1.53^b^	1.82^ab^	0.18	0.1382
	%	23.1^a^	14.8^b^	14.5^b^	1.39	0.0003
Neutrophils	10^3^/mm^3^	6.22^b^	7.89^b^	10.85^a^	0.93	0.007
	%	71.0^b^	76.4^a^	78.7^a^	1.15	0.0003
Monocytes	10^3^/mm^3^	0.36	0.43	0.63	0.13	0.3346
	%	4.13	4.13	4.1	0.66	0.9995
Eosinophils	10^3^/mm^3^	0.12^b^	0.38^a^	0.28^ab^	0.077	0.082
	%	1.34^b^	3.65^a^	1.90^b^	0.56	0.0206
Basophils	10^3^/mm^3^	0.040^b^	0.106^a^	0.095^a^	0.018	0.0403
	%	0.44^b^	1.03^a^	0.71^ab^	0.15	0.033

^ab^Means in a row with different superscripts differ, *P* < 0.05.

WBC, white blood cell count; RBC, red blood cell count; MCV, mean corpuscular volume; MCH: mean corpuscular hemoglobin; MCHC, mean corpuscular hemoglobin concentration.

**Table 5.** Blood chemistry of Labrador Retrievers fed a grain-free diet for 26 wk (*n* = 8)

Blood parameter	Unit	Week			SEM	*P*-value
		0	13	26		
Total protein blood	g/dL	6.29	6.36	6.25	0.13	0.8137
Albumin	g/dL	3.84	3.88	3.7	0.082	0.3048
Globulin	g/dL	2.44	2.46	2.56	0.15	0.8246
A/G ratio		1.59	1.6	1.52	0.1	0.8429
ALT	U/L	54.4^a^	38.1^b^	38.8^b^	4.28	0.0217
Alkaline phosphatase	U/L	40.1^a^	28.6^b^	23.8^b^	2.87	0.0019
Total bilirubin	mg/dL	0.30^b^	0.30^b^	0.36^a^	0.011	0.0004
Creatinine	mg/dL	0.85^b^	0.84^b^	1.14^a^	0.053	0.0008
Blood urea nitrogen	mg/dL	13.1^c^	17.8^b^	22.9^a^	1.34	0.0002
BUN/Creatinine ratio		15.5^b^	22.3^a^	20.2^ab^	1.74	0.0352
Phosphorus	mg/dL	4.16^b^	4.81^a^	4.01^b^	0.17	0.0075
Glucose	mg/dL	105^a^	103^ab^	98^b^	1.97	0.0655
Calcium	mg/dL	10.6	10.5	10.5	0.085	0.5582
Sodium	nmol/L	145^ab^	144^b^	146^a^	0.66	0.0597
Potassium	nmol/L	4.13^b^	4.30^ab^	4.43^a^	0.085	0.0635

^abc^Means in a row with different superscripts differ, *P* < 0.05.

A/G ratio, albumin to globulin ratio; ALT, alanine aminotransferase.

In conclusion, Labradors Retrievers fed a commercial grain-free diet had increased plasma Met, cystine and taurine, and increased whole blood taurine concentrations over a 26-wk feeding study. Urinary taurine:creatinine was not affected throughout the study and fecal excretion of BA increased over time. The current study suggests that the grain-free diet tested does not affect taurine status or gross indicators of health over a 26-wk period. Although dog’s cardiac function was not evaluated in this study, it should be considered in future research.
